# Distribution and Evolution of Nitrogen Fixation Genes in the Phylum *Bacteroidetes*

**DOI:** 10.1264/jsme2.ME14142

**Published:** 2015-01-16

**Authors:** Jun-ichi Inoue, Kenshiro Oshima, Wataru Suda, Mitsuo Sakamoto, Takao Iino, Satoko Noda, Yuichi Hongoh, Masahira Hattori, Moriya Ohkuma

**Affiliations:** 1 Japan Collection of Microorganisms/Microbe Division, RIKEN BioResource CenterKoyadai 3–1–1, Tsukuba, Ibaraki 305–0074Japan; 2 Synaptech Co. Ltd.Ohte 1–2–37–C–105, Kofu, Yamanashi 400–0015Japan; 3 Center for Omics and Bioinformatics, Graduate School of Frontier Sciences, The University of TokyoKashiwanoha 5–1–5, Kashiwa, Chiba 277–8561Japan; 4 Interdisciplinary Graduate School of Medicine and Engineering, University of YamanashiTakeda 4–3–11, Kofu, Yamanashi 400–8511Japan; 5 Department of Biological Sciences, Tokyo Institute of TechnologyOokayama 2–12–1–W3–48, Meguro-ku, Tokyo 152–8550Japan; 6 Biomass Research Platform Team, RIKEN Biomass Engineering Program Cooperation Division, RIKEN Center for Sustainable Resource ScienceKoyadai 3–1–1, Tsukuba, Ibaraki 305–0074Japan

**Keywords:** *nif* gene, nitrogenase, *Bacteroidales*, symbiosis, termite

## Abstract

Diazotrophs had not previously been identified among bacterial species in the phylum *Bacteroidetes* until the rapid expansion of bacterial genome sequences, which revealed the presence of nitrogen fixation (*nif*) genes in this phylum. We herein determined the draft genome sequences of *Bacteroides graminisolvens* JCM 15093^T^ and *Geofilum rubicundum* JCM 15548^T^. In addition to these and previously reported ‘*Candidatus* Azobacteroides pseudotrichonymphae’ and *Paludibacter propionicigenes*, an extensive survey of the genome sequences of diverse *Bacteroidetes* members revealed the presence of a set of *nif* genes (*nifHDKENB*) in strains of *Dysgonomonas gadei*, *Dysgonomonas capnocytophagoides*, *Saccharicrinis fermentans*, and *Alkaliflexus imshenetskii*. These eight species belonged to and were distributed sporadically within the order *Bacteroidales*. Acetylene reduction activity was detected in the five species examined, strongly suggesting their diazotrophic nature. Phylogenetic analyses showed monophyletic clustering of the six Nif protein sequences in the eight *Bacteroidales* species, implying that nitrogen fixation is ancestral to *Bacteroidales* and has been retained in these species, but lost in many other lineages. The identification of *nif* genes in *Bacteroidales* facilitates the prediction of the organismal origins of related sequences directly obtained from various environments.

Biological nitrogen fixation, the conversion of atmospheric dinitrogen to ammonia, has a significant impact on nitrogen cycles in ecosystems. Nitrogen-fixing microorganisms (diazotrophs) are widely distributed in diverse prokaryotic phyla, but sparsely within these phyla. This distribution pattern suggests that nitrogen fixing ability is evolutionary ancient and mainly transmitted vertically with the widespread loss of function (12). The recent rapid expansion of microbial genome sequences has revealed the presence of the genes encoding homologous proteins to known nitrogenases, even in prokaryotic species that had not previously been recognized as diazotrophs (9). In some cases, experimental evidence for nitrogen fixation was obtained following identification of the responsible genes in the genome (23, 25).

The nitrogenase complex consists of its catalytic components encoded by *nifD* and *nifK* and nitrogenase reductase encoded by *nifH*. In addition to ordinary molybdenum-dependent nitrogenases (Nif), vanadium-dependent (Vnf) and iron-only alternative nitrogenases (Anf) have also been identified, but occur in a limited number of diazotrophs. These Nif, Vnf, and Anf types of nitrogenases are homologous and evolutionarily related, except for an additional subunit, VnfG or AnfG, in the Vnf or Anf type, respectively (39). In addition, the factors involving metal cofactor assembly such as those encoded by *nifE*, *nifN*, and *nifB* are necessary for functional nitrogenase. *nifE* and *nifN* are homologous to *nifD* and *nifK*, respectively, and may have originated from ancient gene duplications (5, 10).

The phylum *Bacteroidetes* comprises a huge assemblage of diverse bacterial species isolated from various environments and have been classified into four orders (according to the list of prokaryotic names with standing in nomenclature; http://www.bacterio.net/). The order *Bacteroidales* in this phylum comprises nearly 40 genera and many species in this order are Gram-negative anaerobic rods that are frequently isolated from human and animal gastrointestinal tracts. Although a large number of genome-sequenced strains exist in this order, many are isolates from human specimens.

Diazotrophs had not previously been identified among members in the phylum *Bacteroidetes*; however, a recent genome survey revealed the presence of nitrogenase homologous genes. The first genome of the *Bacteroidetes* member that encodes *nif* genes was reported in ‘*Candidatus* Azobacteroides pseudotrichonymphae’, an abundant endosymbiont *Bacteroidales* species of a cellulolytic protist in the gut of the termite *Coptotermes formosanus* (16). In that study, it was hypothesized that *nif* genes had been acquired via lateral gene transfer from a bacterium co-inhabiting the gut. *nif* genes were also detected in the genome sequence of another *Bacteroidales* member, *Paludibacter propionicigenes* strain WB4T (9). These findings suggested that *nif* genes are more widespread, but sporadic among *Bacteroidetes* members than currently recognized. No direct experimental evidence currently exists for the nitrogen fixing abilities of these two species.

In the present study, the homologous sequences of *nif* genes were surveyed in the genomes of diverse *Bacteroidetes* members. Based on the presence of a set of *nif* genes and results of the acetylene reduction assay, we identified several *Bacteroidales* members as new potential diazotrophs. Phylogenetic analyses suggested that the *nif* genes of the identified species have been vertically transmitted from a common ancestor. The identification of potential diazotrophs in *Bacteroidales* may have an impact on their ecology and their genes can be useful references for metagenomic studies on microbial communities.

## Materials and Methods

### Genome survey of *nif* genes

The genome sequences of bacterial strains in *Bacteroidetes* were retrieved from the DDBJ database as of July 1st, 2013 (108 genomes) and August 13th, 2014 (an additional 1,238 genomes). The draft genome sequences of 62 strains of *Bacteroidetes* determined in our laboratories were included in these genomes (for the list of strains and BioProject ID, see http://jcm.brc.riken.jp/en/nbrplist_e). The *nif* genes were initially searched in these genome sequences with BLAST (1) at a relatively low-level threshold, e-value >10E-20, using *nifH* and *nifD* of ‘*Ca.* A. pseudotrichonymphae’ (locus tags CFPG_545 and CFPG_548, respectively; database accession number AP010656) as queries, and then inspected manually with phylogenetic analyses (see below). This low-level threshold ensured the detection of distantly related sequences even in the outside groups of uncharacterized *nif*-like sequences. The presence of other *nif* genes was examined in the selected genome sequences based on the existing annotations and further BLAST searches. The genome sequence of *P. propionicigenes* WB4^T^ (database accession number CP002345) (11) was included in the analyses as a reference.

### Draft genome sequencing

All the bacterial strains used in this study were provided by the Japan Collection of Microorganisms (JCM). These strains were cultured with the conditions specified according to the JCM online catalog database (http://jcm.brc.riken.jp/en/catalogue_e). The draft genome sequences of *Bacteroides graminisolvens* JCM 15093^T^ and *Geofilum rubicundum* JCM 15548^T^ were determined, assembled, and annotated with the methods described previously (48).

### Acetylene reduction assay

Bacterial strains were cultured with the JCM-specified, nutrientrich medium and the mass of the cultured cells was then inoculated to the nitrogen-poor N_2_ -fixation medium in a 50-mL stopper bottle. One liter of the N_2_ -fixation medium comprised 0.688 g of K_2_ HPO_4_ , 0.19 g of Na_2_ SO_4_ ·10H_2_ O, 3.75 g of CaCO_3_ , 30 g of sucrose, a trace amount of biotin, and 1 mL of mineral solution. One liter of the mineral solution comprised 2.4 g of NaMoO_4_ ·2H_2_ O, 0.24 g of CoCl_2_ ·6H_2_ O, 1.5 g of CaCl_2_ ·2H_2_ O, 27 g of FeCl_3_ ·6H_2_ O, 28 mL of H_2_ SO_4_ , 0.25 g of CuSO_4_ ·5H_2_ O, 0.29 g of ZnSO_4_ ·7H_2_ O, 1.7 g of MnSO_4_ ·H_2_ O, and 12 g of MgSO_4_ . The gas phase of the medium was initially replaced with N_2_ and then with 30% of acetylene. After being incubated for 7 d, a 0.1-mL gas sample was assayed after 100-fold dilution with N_2_ for ethylene production using a gas chromatograph (GC-2014ATC, Shimadzu, Kyoto, Japan) with the Porapak T (80/100 mesh) column (GL Science, Tokyo, Japan) and flame ion detector operating at 50°C and 85°C, respectively. The sensitivity of the measurements was sufficient, even after the dilution. The carrier gas was N_2_ at a flow rate of 30 mL min^−1^. The bacterial cell numbers of the cultures were estimated as the most probable numbers.

### Phylogenetic analyses

*In silico* translated amino acid sequences were aligned using MAFFT 7 (22) and manually refined. Only unambiguously aligned residues were used in phylogenetic analyses. Maximum likelihood trees were inferred with RaxML MPI version 8.1.2 (41) using the best model selected with Aminosan in the Kakusan4 package (44). A concatenate sequence analysis was also conducted using the best model for each protein and optimizing the parameter in each protein. Bootstrap analyses of 1,000 replicated re-samplings were conducted to estimate confidence for tree topologies.

### Sequence accession numbers

The draft genome sequences of *B. graminisolvens* JCM 15093^T^ and *G. rubicundum* JCM 15548^T^ have been deposited in DDBJ/EMBL/GenBank under accession numbers BAJS00000000 and BAZW00000000, respectively.

## Results and Discussion

### Genome survey of the *nif* gene

A total of 1,346 genome sequences of *Bacteroidetes* strains were searched for among homologous genes encoding conventional nitrogenases. In addition to ‘*Ca.* A. pseudotrichonymphae’ and *P. propionicigenes* WB4^T^, homologous genes were detected in the genomes of *Dysgonomonas gadei* ATCC BAA-286^T^ (ADLV00000000, unpublished), *Saccharicrinis fermentans* JCM 21142^T^ (BAMD00000000 [43]; recently renamed from *Cytophaga fermentans* [47]), *Bacteroides graminisolvens* JCM 15093^T^, and *Geofilum rubicundum* JCM 15548^T^. The homologous genes were also detected in the very recently appeared genomes of *Dysgonomonas capnocytophagoides* DSM 22835^T^ (NZ_AUFL00000000; unpublished) and *Alkaliflexus imshenetskii* DSM 15055^T^ (AJUM00000000, unpublished); these two strains were analyzed phylogenetically with their *nif* genes, but were not assayed for acetylene reduction. The frequency of the *nif* genes was only 0.5% among the searched genomes. One possible reason for this low frequency is that many genome sequences are still determined with strains associated with humans or animals, and this habitat is likely to be rich in available nitrogen sources.

All these species belong to the order *Bacteroidales* (class *Bacteroidia*), and are distributed in three families, *Marinilabiliaceae* (*Saccharicrinis*, *Geofilum*, and *Alkaliflexus*), *Porphyromonadaceae* (*Dysgonomonas* and *Paludibacter*), and *Bacteroidaceae* (*Bacteroides*), among the six recently updated families within *Bacteroidales* (20). The gene encoding a homologous protein to conventional nitrogenases was not detected in the genome sequences of strains in the other classes in *Bacteroidetes* (*Cytophagia*, *Flavobacteriia*, and *Sphingobacteriia*), although they accounted for 42.6% of the searched genomes. The isolation sources of these potential diazotrophic species were diverse; human clinical specimens for two *Dysgonomonas* species (14), rice plant residue in anoxic rice-field soil for *P. propionicigenes* (45), rice straw residue in a methanogenic reactor for *B. graminisolvens* (27), deep subsea floor sediment for *G. rubicundum* (26), marine mud for *S. fermentans* (4), and an alkaline soda lake for *A. imshenetskii* (50). They presumably maintained their nitrogen fixation ability for their ecological demands. These isolation sources were not always very poor in available nitrogen; however, if these potential diazotrophs share other habitats poor in nitrogen sources, nitrogen fixation may be of significant importance for their survival and adaptation.

### Annotation of draft genomes of *B. graminisolvens* and *G. rubicumdum*

The draft genome sequences of *B. graminisolvens* JCM 15093^T^ and *G. rubicumdum* JCM 15548^T^ were assembled and annotated in the present study. The total sequence reads of 620,620 for *B. graminisolvens* and 553,196 for *G. rubicumdum* were assembled into 63 and 212 contigs with N_50_ lengths of 128,246 bp and 65,843 bp, 39.0 and 23.4×redundancies, and G+C contents of 41.6% and 44.8%, respectively. The resulting genomes of 3.68 Mbp for *B. graminisolvens* and 4.92 Mbp for *G. rubicumdum* contained 3,413 and 4,556 protein coding sequences, respectively.

### Identification of functional *nif* genes

**Fig. 1:**
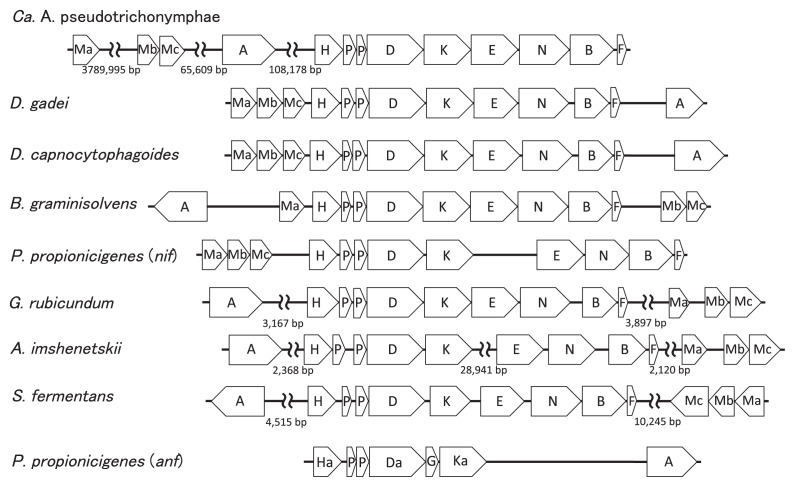
Structure of the *nif* gene cluster in genomes of *Bacteroidales* species. The genes indicated by H, D, K, E, N, B, and A are *nifH*, *nifD*, *nifK*, *nifE*, *nifN*, *nifB*, and *nifA*, respectively. Genes indicated by P are *glnB*-like putative regulators for nitrogen fixation, those indicated by F are ferredoxin-encoding genes, and those indicated by Ma, Mb, and Mc are *modA*, *modB*, and *modC* homologous genes, respectively, for molybdenum availability. In *P. propionicigenes*, *anfH*, *anfK*, *anfG*, and *anfD* are indicated by Ha, Ka, Ga, and Da, respectively. When the genes are located in separate genome regions, the distance between genes are indicated.

Annotations of the genome sequences revealed the presence of *nifH*, *nifD*, *nifK*, *nifE*, *nifN*, and *nifB* in the eight *Bacteroidales* genomes. These six genes (*nifHDKENB*) were clustered in this gene order in the seven genomes, except for *A. imshenetskii* DSM 15055^T^ (Fig. 1). In *A. imshenetskii*, *nifHDK* and *nifENB* were separated by a distance of 29 kb. These six genes corresponded to the minimum *nif* gene set proposed as a criterion for predicting nitrogen fixation based on genome sequences (9).

In every genome, the ferredoxin-encoding gene was located downstream of *nifB*, and two small genes homologous to *glnB* existed between *nifH* and *nifD*, which may be involved in the regulation of *nif* gene expression (2). The transcriptional regulator gene *nifA* was sometimes located near the *nifHDKENB* gene cluster. The genes responsible for molybdenum availability such as *modA*, *modB*, and *modC* were also detected near this gene cluster, but their gene order and relative location to the other genes were not always conserved.

The presence of alternative nitrogenase (*anf*) genes was previously reported in the genome of *P. propionicigenes* WB4^T^ (9). In this genome, the *anf* gene cluster comprised *anfHDGK* and two *glnB* homologous genes present between *anfH* and *anfD*. The *anfHDGK* and *nifHDKENB* clusters were located distantly to each other in the genome (49 kb distance). In our survey of genome sequences, *P. propionicigenes* WB4^T^ was the only *Bacteroidetes* member that had the *anf* gene.

### Acetylene reduction activity

Nitrogen fixation ability was examined in five strains that harbored the set of *nif* genes described above. Acetylene reduction activity was measured for this purpose because this activity was very sensitive and widely used to measure nitrogenase activity. In all of the five examined strains, significant activity, which corresponded to 1/8 to 1/25 that of the diazotrophic strain *Clostridium pasteurianum* JCM 1408^T^, was detected after the culture was shifted to the nitrogen poor medium (Table 1). Together with the presence of the *nif* gene set, these results strongly suggested that these five strains had the ability to fix dinitrogen.

Although evaluating ^15^N_2_ stable isotope incorporation is important for providing more direct evidence for nitrogen fixation, the acetylene reduction activities detected were very low and isotope incorporation was not expected. Prominent growth on the nitrogen-poor medium used in this study was not detected for any species, and this may have been because the medium lacked some essential nutrients. The optimization of culturing conditions is necessary in order to further characterize the nitrogen fixation abilities of these species.

### Phylogeny of *nif* gene sequences

**Fig. 2:**
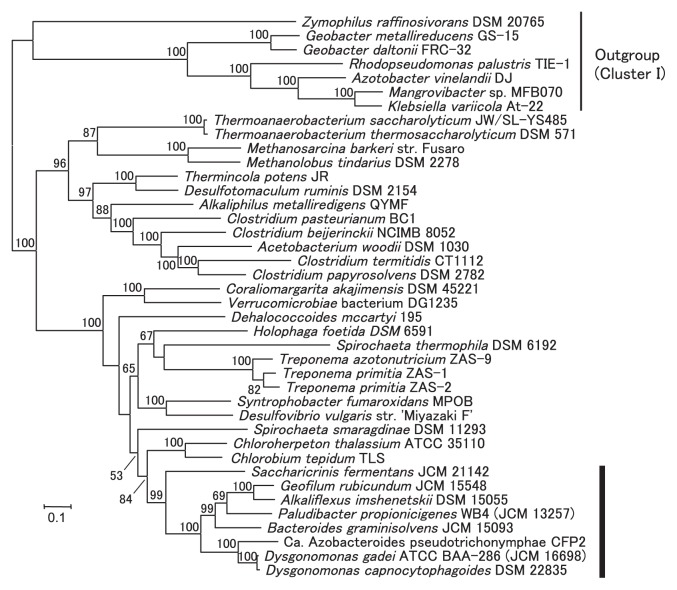
Phylogenetic relationships of potential nitrogen-fixing species carrying the Cluster III of nitrogenases inferred based on the sequence concatenation of six proteins NifH, NifD, NifK, NifE, NifN, and NifB. A total of 1,999 amino acid sites were used to construct the maximum likelihood tree with the selected best model for each protein: LG+G+I+F for NifH, NifD, and NifB, and LG+G+I for NifK, NifE, and NifH. The thick vertical bar indicates the monophyletic clade of the *Bacteroidales* species. Nitrogen-fixing species carrying Cluster I of nitrogenases were used as outgroups (indicated with the thin vertical bar). Bootstrap values in percentages are indicated at the nodes when the values were over 50%. The scale bar corresponds to 0.1 substitutions per site.

In the phylogenetic tree inferred with the concatenated sequences of the six proteins NifH, NifD, NifK, NifE, NifN, and NifB (Fig. 2), the eight species of *Bacteroidales* formed a monophyletic cluster in Group III of the nitrogenases defined by Raymond *et al.* (39) with strong bootstrap support (99%). This result implied that an ancestor of *Bacteroidales* had the set of *nif* genes that had been vertically transmitted during their evolution. If this was the case, the sporadic occurrence of *nif* genes in *Bacteroidales* members can be attributed to the loss of *nif* genes in many lineages. The nitrogen fixation reaction requires a large amount of energy, and once species adapted to environments rich in available nitrogen sources, they may have lost the *nif* genes. The sporadic occurrence of diazotrophs is a common feature among other prokaryotic phyla.

Among the phylogenetic trees of the individual protein sequences (Figs. S1–S6), the monophyly of the eight species occurred in NifK, NifE, and NifB with fairly strong support in the former two (78% and 89%, respectively), but with very weak support in the latter (41%). *S. fermentans* was located outside the strongly supported monophyletic group of the other seven species in the NifH and NifD trees, whereas *S. fermentans* branched out very closely and its position was only weakly supported. Three taxa of the genus *Treponema* were branched within the group of the eight species in the NifN tree; however, these relationships were very weakly supported.

The relationships among the *Bacteroidales* members were almost fully resolved in the concatenated tree (Fig. 2) and many of these relationships were also observed in the single protein trees (Fig. S1–S6). Except in the NifH tree, ‘*Ca.* A. pseudotrichonymphae’ and the two *Dysgonomonas* species were always grouped together with strong support (>99%). ‘*Ca.* A. pseudotrichonymphae’ and *Dysgonomonas* species were found to be closely related to each other in an analysis of multiple proteins (M. Yuki, unpublished data), but were distantly related in the 16S rRNA gene sequence analyses reported previously (7, 15, 30, 33). *A. imshenetskii* and *G. rubicundum* were sisters to each other in every tree and this relationship was supported fairly in NifH (82%) and strongly in the other trees (100% each). These two species belong to the family *Marinilabiliaceae*; however, *S. fermentans*, which belongs to the same family, did not group together in any of the trees. The two *Dysgonomonas* species and *P. propionicigenes*, both of which belong to the family *Porphyromonadaceae*, were also not grouped together, except in the NifK tree. Vertical transmission from a common ancestor may be a general rule for the *Bacteroidales nif* genes, but not the sole rule for their evolution because strict vertical transmission cannot explain the observed phylogenetic relationship.

### Related *nif* gene sequences in environments

The identification of *nif* genes in *Bacteroidales* members facilitates the prediction of organismal origins of related sequences obtained directly from various environments. The *nifH* sequences were used as queries for searches of related sequences in the DNA sequence database because *nifH* is often used to detect potential nitrogen fixers in various environments (49). These searches exclusively identified a large number of *nifH* sequences from the microbial community in the gut of termites as closely related sequences. The sequences from other environments showed less than 90% amino acid identity to the NifH protein sequence of ‘*Ca.* A. pseudotrichonymphae’.

Termites thrive on nitrogen-poor dead plant materials, and besides cellulose decomposition, nitrogen fixation by the gut bacteria is another crucial aspect of symbiosis with the gut microbial community (6). The diversity of *nifH* sequences was previously investigated in various termite species and the related cockroach *Cryptocercus punctulatus* (29, 34, 35, 46), and the relationships between *nifH* diversity in the gut microbial communities and the lifestyles and phylogenetic positions of host termites have been inferred (46). Although the microorganisms encoding most of these *nifH* sequences are unknown, the identification of *nif* genes in *Treponema* species (phylum *Spirochaetes*) isolated from a termite gut has enabled us to predict the *Treponema* origins of some *nifH* sequences (24). *Treponema* and *Bacteroidales* members are major constituents of gut microbial communities and many species, such as ‘*Ca.* A. pseudotrichonymphae’, are associated with gut cellulolytic protists as endo- or ectosymbionts (17, 37). Bacterial species in *Bacteroidales* may play a crucial role in symbiotic nitrogen fixation because the *nifH* and *anfH* genes related to the *Bacteroidales* members are some of the most abundant sequences detected in the gut microbial communities of many termite species.

The *nifH* sequences of *Bacteroidales* members were closely related to a group of sequences in “Cluster III-1” defined by Yamada *et al.* (46), and corresponded to the “Bact III-3a” cluster recently defined by Desai and Brune (8), which includes the *nifH* sequences identified from protist suspensions and predicted to be derived from the ectosymbiont species ‘*Candidatus* Armantifilum devescovinae’. ‘*Ca.* A. devescovinae’ and related ectosymbiont species of termite-gut protists, together with the ‘*Ca.* A. pseudotrichonymphae’ endosymbiont, belong to Cluster V of *Bacteroidales*, defined based on the 16S rRNA gene sequence (7, 30–33, 36). Therefore, these findings confirmed the ‘*Ca.* A. devescovinae’ origin of the sequences identified from protist suspensions. The sequences of *D. gadei*, *D. capnocytophagoides*, and *B. gramnisolvens* were also closely related to the sequences from the termite gut, suggesting the presence of related diazotrophic species in addition to species in the Cluster V of *Bacteroidales*.

The *P. propionicigenes anfH* sequence was closely related to the sequences represented by the phylotype Nk09 from the termite gut (35, 46) and sequences identified from the protist suspension harboring ‘*Ca.* A. devescovinae’ (8). These *anfH* sequences were obtained abundantly from termite species belonging to *Kalotermitidae* (so-called dry-wood termites), such as *Neotermes koshunensis*, and the *Cryptocercus* cockroach. Although Desai and Brune (8) previously reported that this *anfH* group of sequences may have been due to secondary acquisition in the lineage including ‘*Ca.* A. devescovinae’, the close relationship with *P. propionicigenes anfH* strongly suggested vertical transmission of the *anf* gene from a common ancestor and the loss of *anf* genes in other lineages such as ‘*Ca.* A. pseudotrichonymphae’. The preferentially transcribed nature of this group of *anf* genes in some *Kalotermitidae* termites (8, 28) can explain the ecological necessity for the retention of the *anf* genes. *P. propionicigenes anfD* and *anfG* also showed high-level amino acid sequence identities to those identified as the preferentially transcribed *anf* genes in *N. koshunensis* (82% and 65% identities, respectively). In addition to the sequences from the termite guts, several unpublished *nifH* sequences from rice roots (*e.g.* AB184916) and activated sludge (*e.g.* AB827428) were closely related to *P. propionicigenes anfH*, suggesting the presence of related species of *Bacteroidales* as diazotrophs in these environments.

### Uncharacterized *nif*-like genes

In our survey of *nif* genes in *Bacteroidetes* genomes, genes encoding proteins slightly homologous, but distantly related to conventional nitrogenases were also found in the genomes of *Prevotella bryantii* B14^T^ (ADWO00000000) (38) and *Bacteroides reticulotermitis* JCM 10512^T^ (BAIV00000000) (48). Detailed phylogenetic analyses indicated that their *nifD* and *nifK* homologs belonged to so-called *nifE*-like and *nifN-*like sequences, respectively, as defined by Dos Santos *et al.* (9) (see Fig. S7 and S8). These NifE-like and NifN-like proteins are considered to have an as yet unknown function, but not in nitrogen fixation. In these NifE-like and NifN-like sequences, the conserved histidine residue (His 442 in the *Azotobacer vinelandii* NifD numbering), required for the ligand of iron-molybdenum co-factor co-ordination in conventional nitrogenases (21), was not found despite the presence of the conserved cysteines that coordinate iron-sulfur clusters. Therefore, *P. bryantii* and *B. reticulotermitis* were unlikely to have nitrogen fixation abilities.

Furthermore, the *nifH* homologous gene found in *B. reticulotermitis* was assigned to the Pseudo-*nif* group (35) or Group IV (39) of *nifH* sequences that was also very unlikely to function as nitrogenases. In contrast, *P. bryantii* had the *nifH* gene sequence closely related to conventional nitrogenases in Group III and the closest relative was that of *Treponema bryantii* (data not shown). Both *nifE*-like and *nifN*-like sequences of *P. bryantii* were closely related to those of *Fibrobacter succinogenes*. Since *P. bryantii*, *T. bryantii*, and *F. succinogenes* are all inhabitants of the rumen of ruminant animals (3, 19, 42), lateral gene transfer may have occurred during their evolution. One of the close relatives of the *nifE*-like or *nifN*-like sequences of *B. reticulotermitis* was that from *Clostridium termitidis*, and both species were isolated from the gut of termites (13, 40), again implying lateral gene transfer. The clone sequence GFN19 in Pseudo-*nif* Cluster I from the termite gut (35) and the three clone sequences Cp08, Cp26, and Cp32 from the *Cryptocercus* cockroach (46) were closely related to the *nifH-*like sequence of *B. reticulotermitis*. These clone sequences may have been derived from related species; however, potential lateral gene transfers need to be considered in this prediction.

## Conclusion

**Fig. 3:**
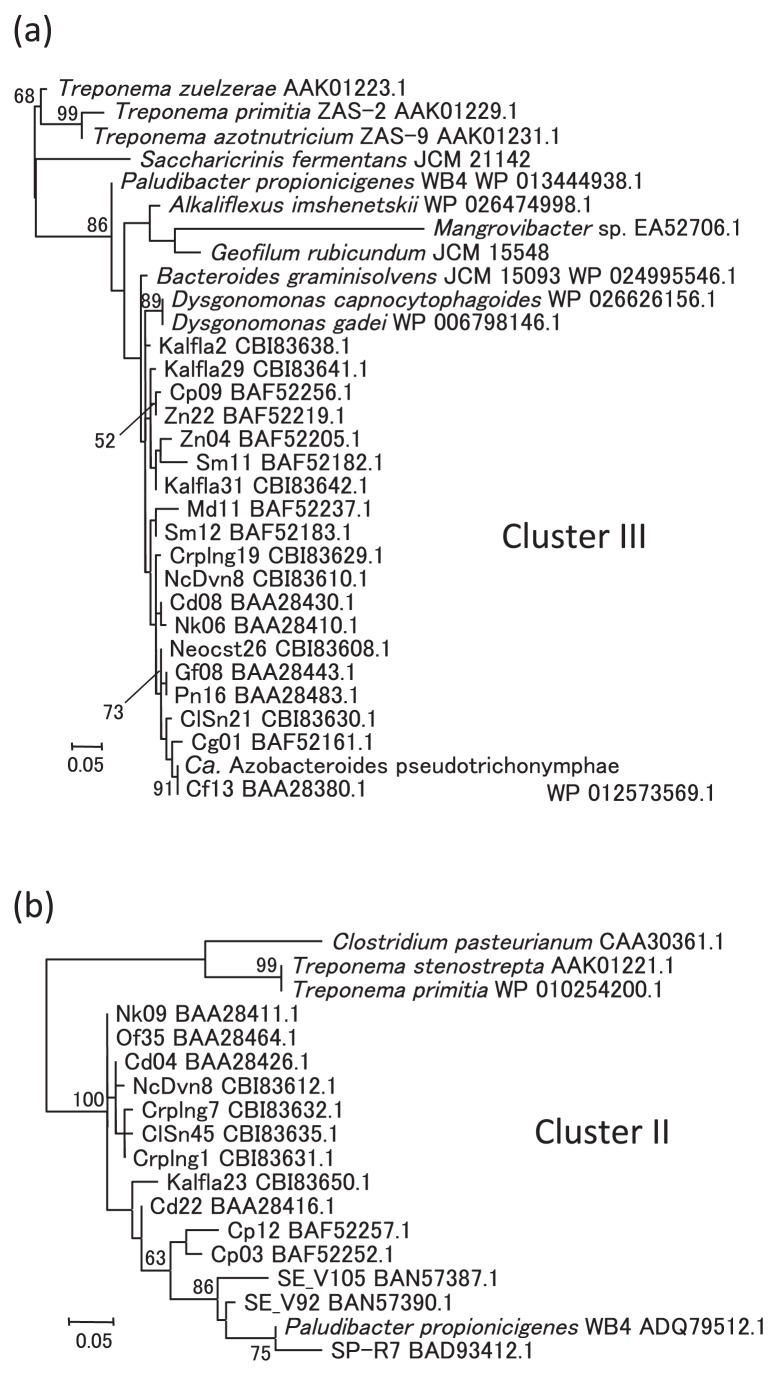
Phylogenetic relationships of NifH protein sequences of *Bacteroidales* species and related sequences from termite-gut microbial communities. The maximum likelihood trees of the Cluster III of NifH (a) and Cluster II of AnfH (b) were inferred based on 141 and 123 amino acid sites with the WAG+G+I model, respectively. Bootstrap values in percentages are indicated at the nodes when the values were over 50%. The scale bar corresponds to 0.1 substitutions per site.

The presence of the set of *nif* genes related to conventional nitrogenase and acetylene reduction under nitrogen poor conditions were strong indications of nitrogen fixation ability in the *Bacteroidales* species identified in this study. The further expansion of potential diazotrophs is expected due to the great species diversity that has not yet been examined by genome sequencing. A related partial *nifH* gene sequence was recently identified in another *Bacteroidales* member, *Mangrovibacterium diazotrophium* (18) (see Fig. 3). As shown in this study for the gut symbiotic community of termites, the identification of *nif* genes in wider varieties of species will be very useful for predicting the more organismal origins of *nif* gene sequences in various environments, which is important for obtaining a clearer understanding of the ecology of diazotrophs and their roles in ecosystems.

## Supplementary Information



## Figures and Tables

**Table 1:** Acetylene reduction activity of *Bacteroidales* species

Strain	Activity (nmol cell^−1^)
*Dysgonomonas gadei* JCM 16698^T^	1.33±0.17×10^−6^
*Bacteroides graminisolvens* JCM 15093^T^	0.08±0.05×10^−6^
*Paludibacter propionicigenes* JCM 13257^T^	1.50±0.20×10^−6^
*Geofilum rubicundum* JCM 15548^T^	0.48±0.04×10^−6^
*Saccharicrinis fermentans* JCM 21142^T^	0.59±0.04×10^−6^
*Clostridium pasteurianum* JCM 1408^T^	12.7±0.04×10^−6^

*C. pasteurianum* JCM 1408^T^ was used as a positive control. Only this strain showed prominent cell growth in the N_2_ fixation medium. Activity was measured after seven days incubation and expressed as the mean ± standard deviation of three replicated measurements. No activity was detected when *Escherichia coli* DH5α, *Dysgonomonas hofstadii* JCM 17038^T^, and *Prevotella paludivivens* JCM 13650^T^ were used as negative controls for measurements.
