# Supplementary material for: Distribution and Evolution of Nitrogen Fixation Genes in the Phylum Bacteroidetes
Source: Microbes Environ. 2015 Jan 16;30(1):44–50. doi: 10.1264/jsme2.ME14142 (PMC4356463; doi:10.1264/jsme2.ME14142)
Supplement: Supplementary file 1 [file 30_44_s1.pdf]

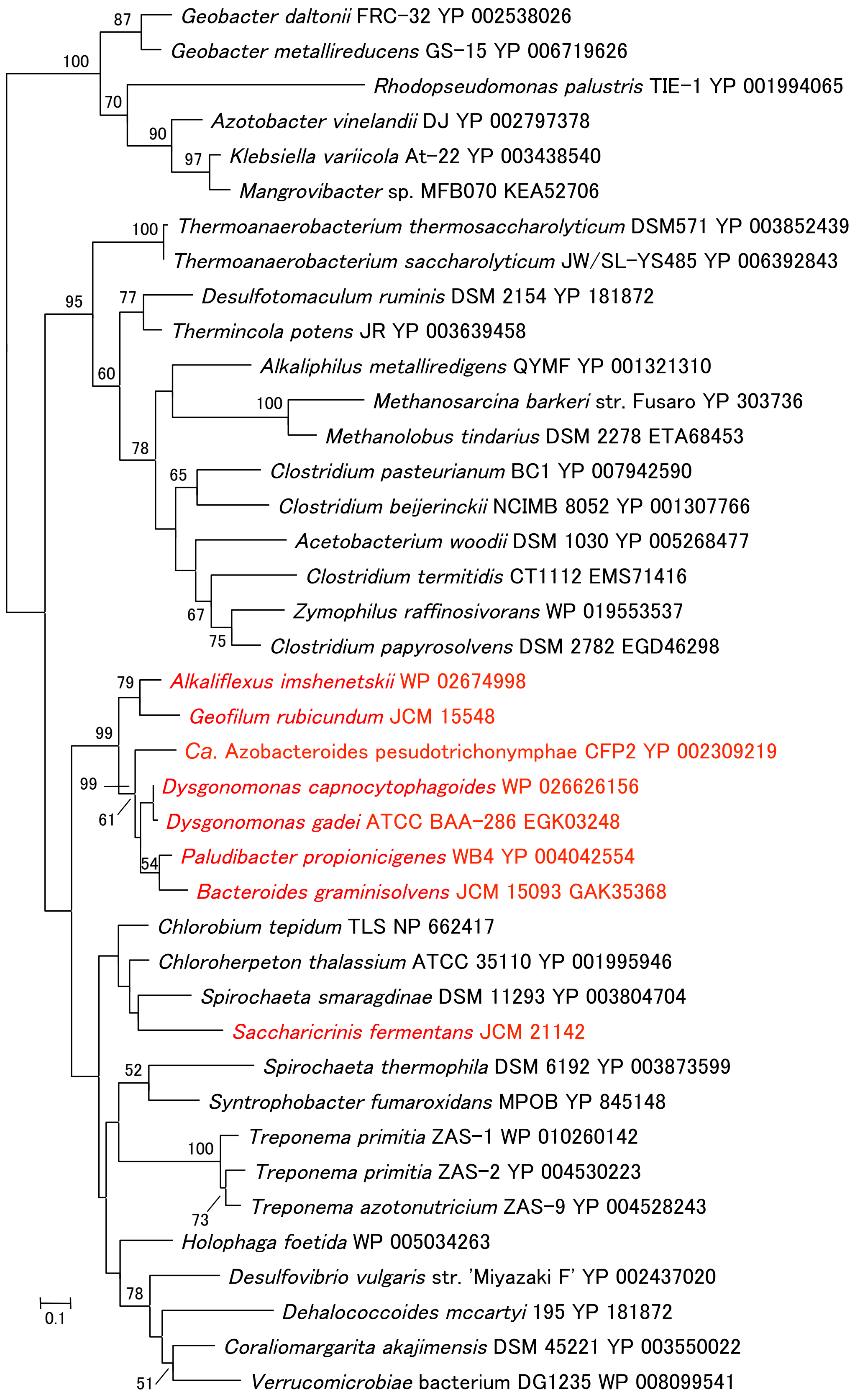

Fig. S1. NiH phylogeny base on 268 amino acid sites. Details are as given in figure 2 in the text.

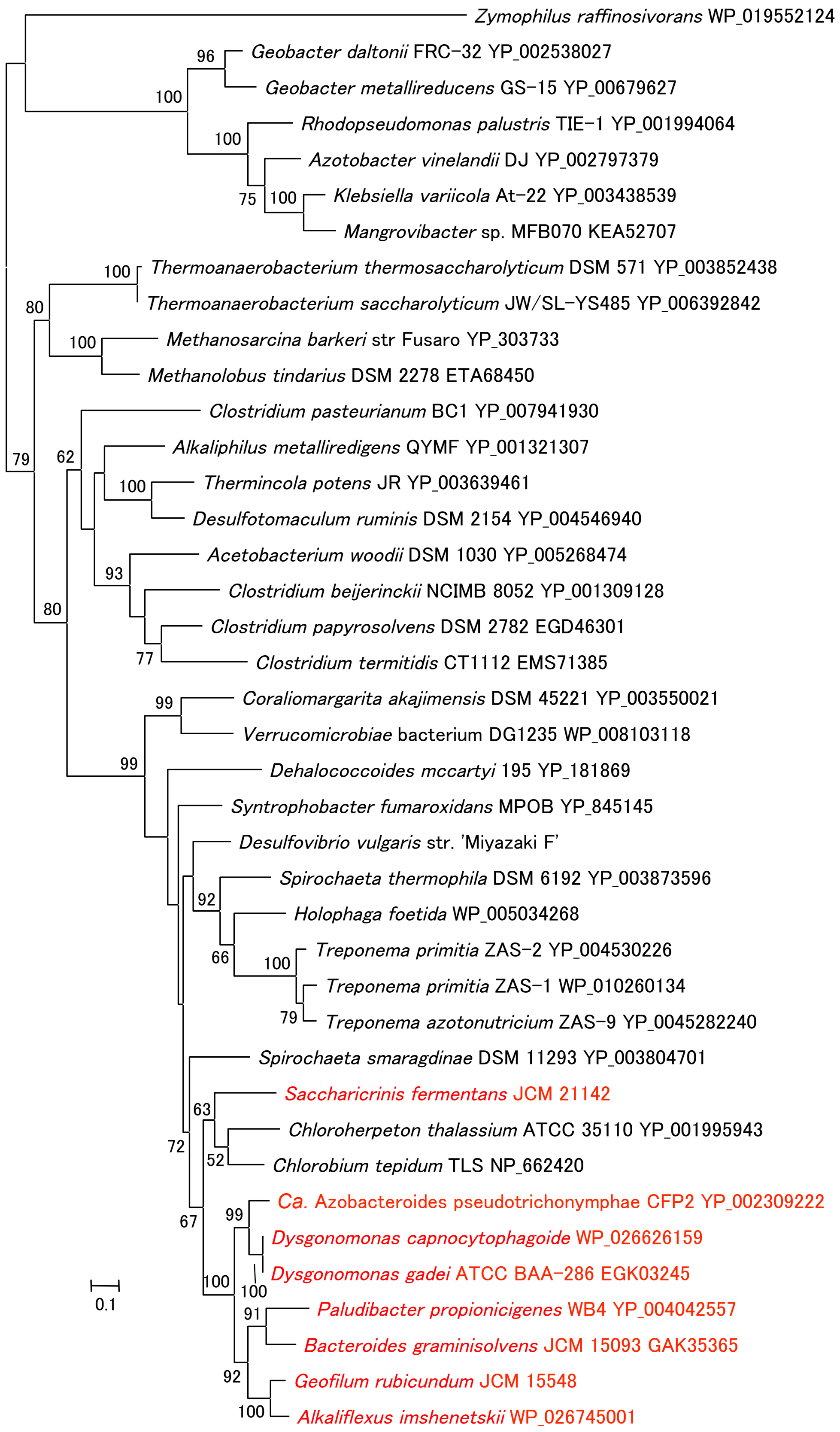

Fig. S2. NifD phylogeny base on 425 amino acid sites. Details are as given in figure 2 in the text.

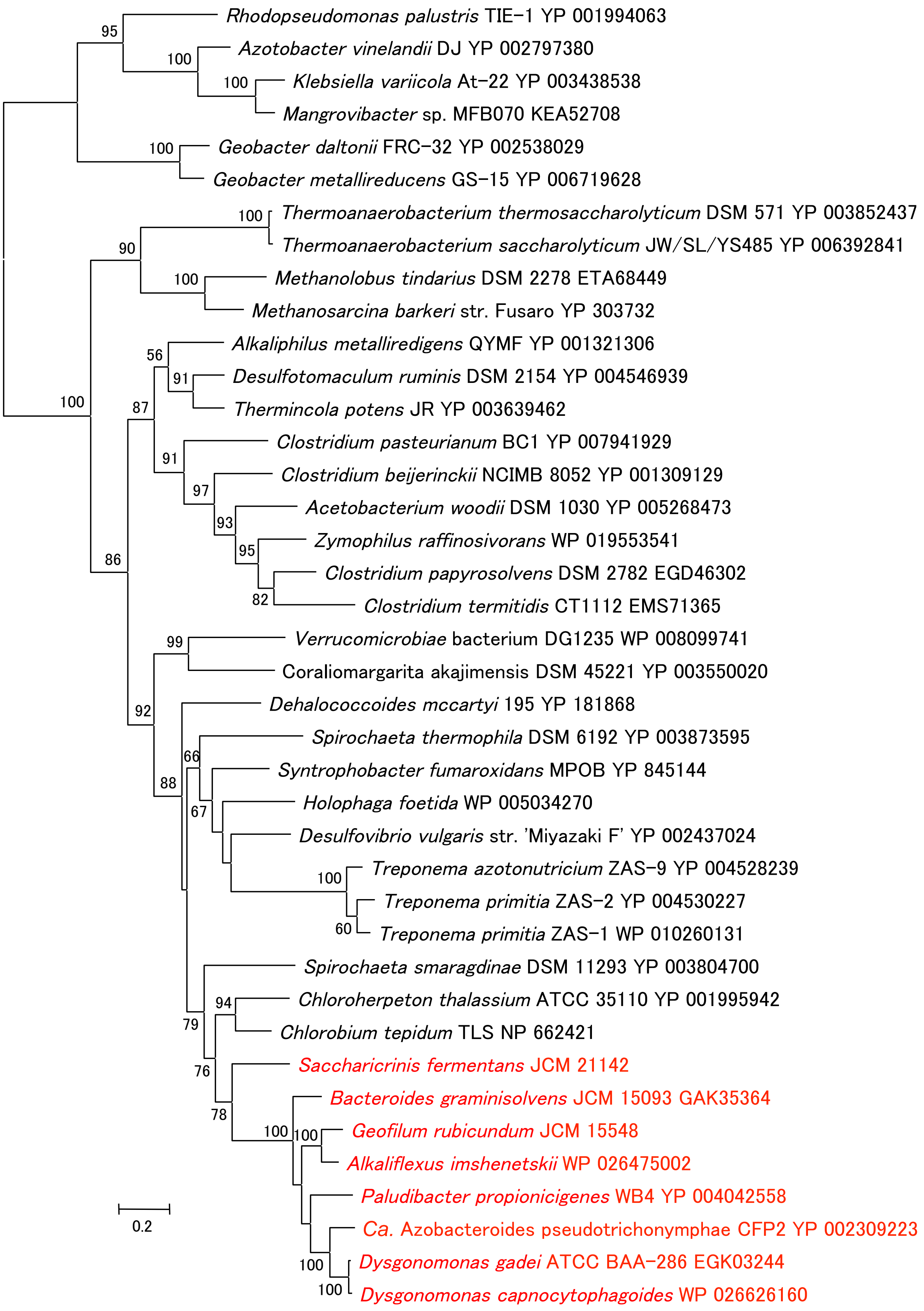

Fig. S3. NifK phylogeny base on 400 amino acid sites. Details are as given in figure 2 in the text.

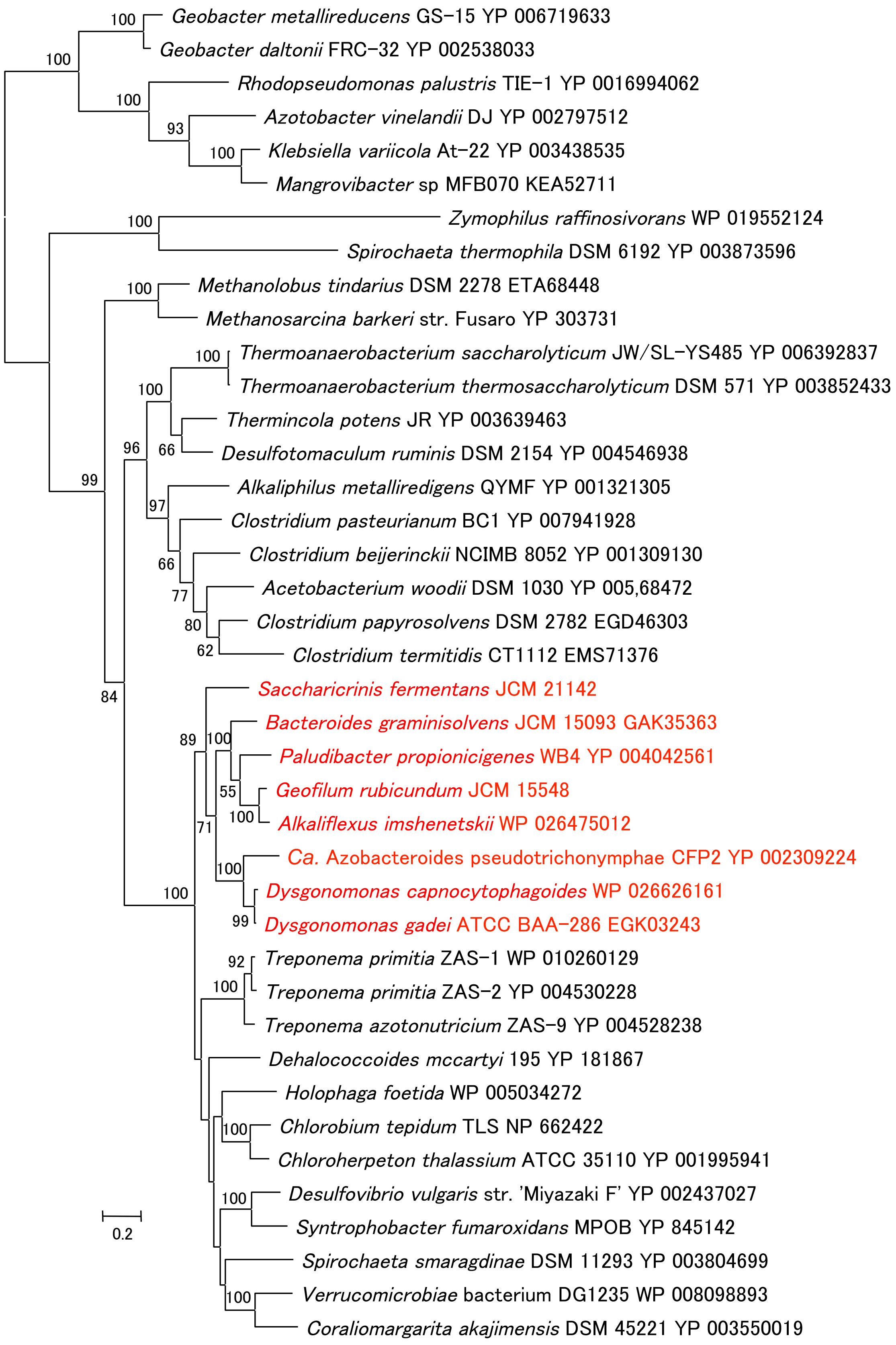

Fig. S4. NifE phylogeny base on 414 amino acid sites. Details are as given in figure 2 in the text.

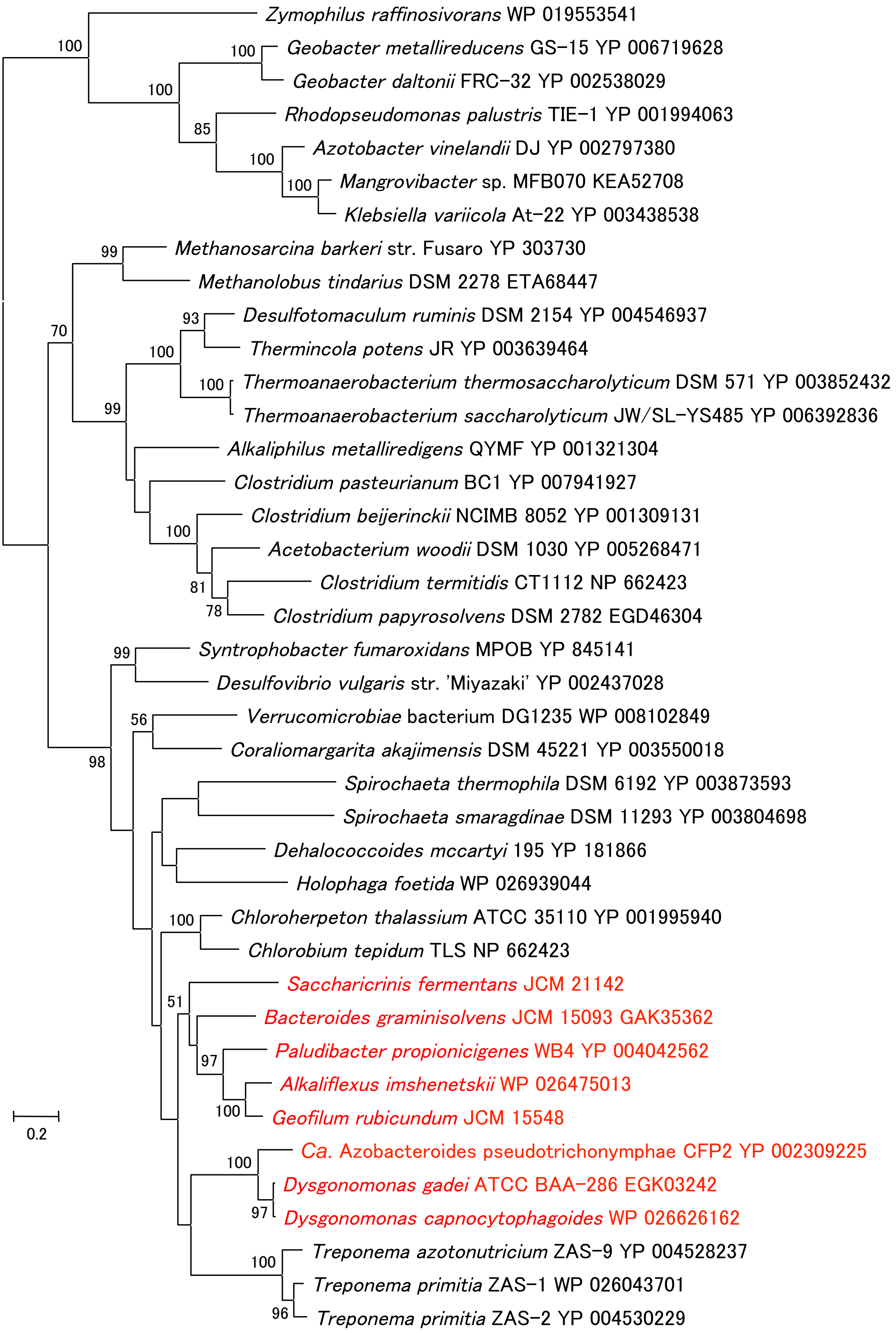

Fig. S5. NifN phylogeny base on 355 amino acid sites. Details are as given in figure 2 in the text.

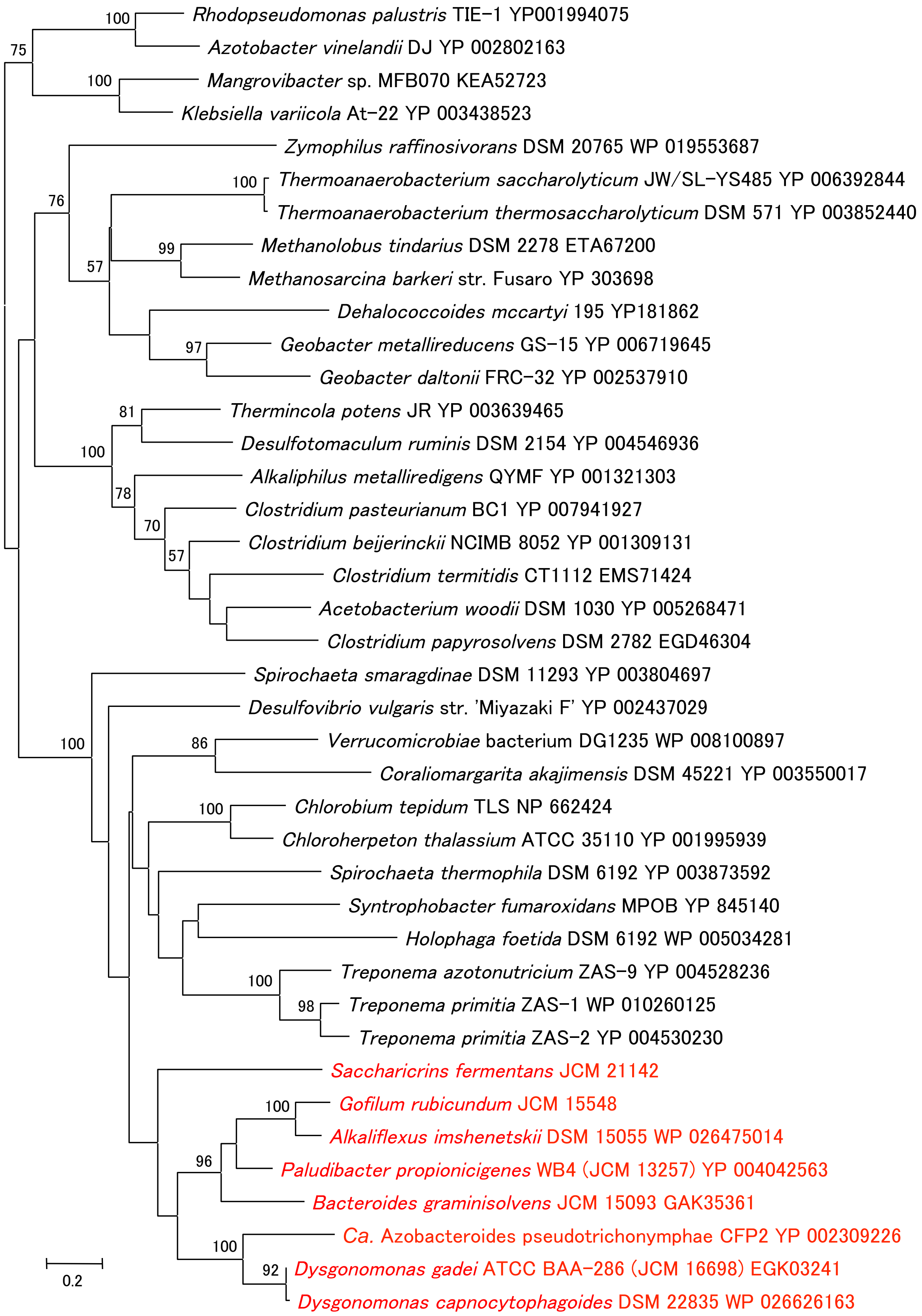

Fig. S6. NifB phylogeny base on 169 amino acid sites. Details are as given in figure 2 in the text.

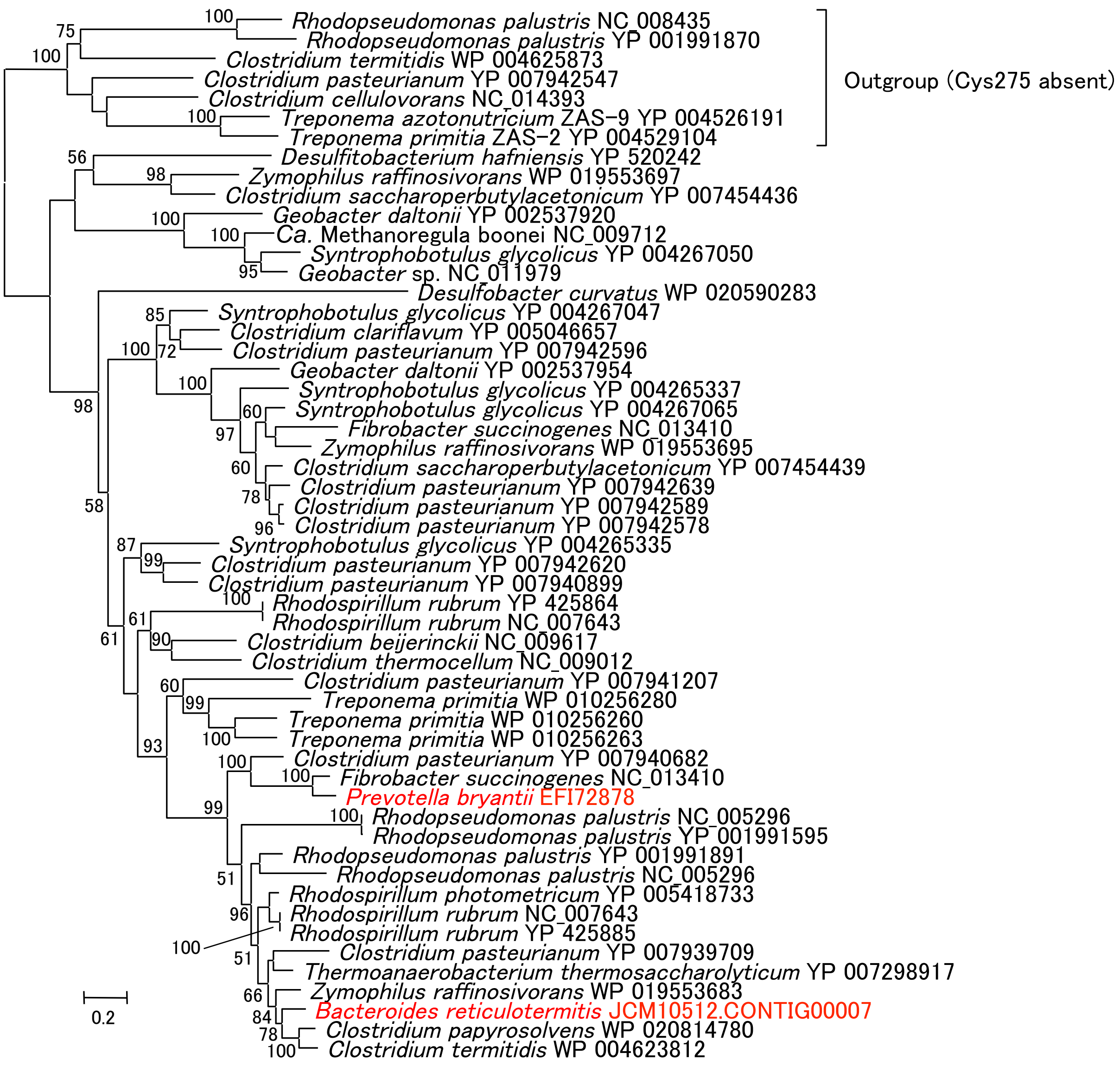

Fig. S7. Maximum likelihood phylogenetic tree of NifE-like sequences. The tree was inferred based on 367 amino acid sites with the selected LG+G+F model. The related sequences in which the iron-molybdenum cofactor ligand Cys 275 (*A. vinelandii* NifD numbering) is absent were used as outgroups. Numbers at nodes indicate bootstrap support values in percentage, when the value is over 50%, with 1000 replicates. The scale bar corresponds to 0.1 substitutions per site.

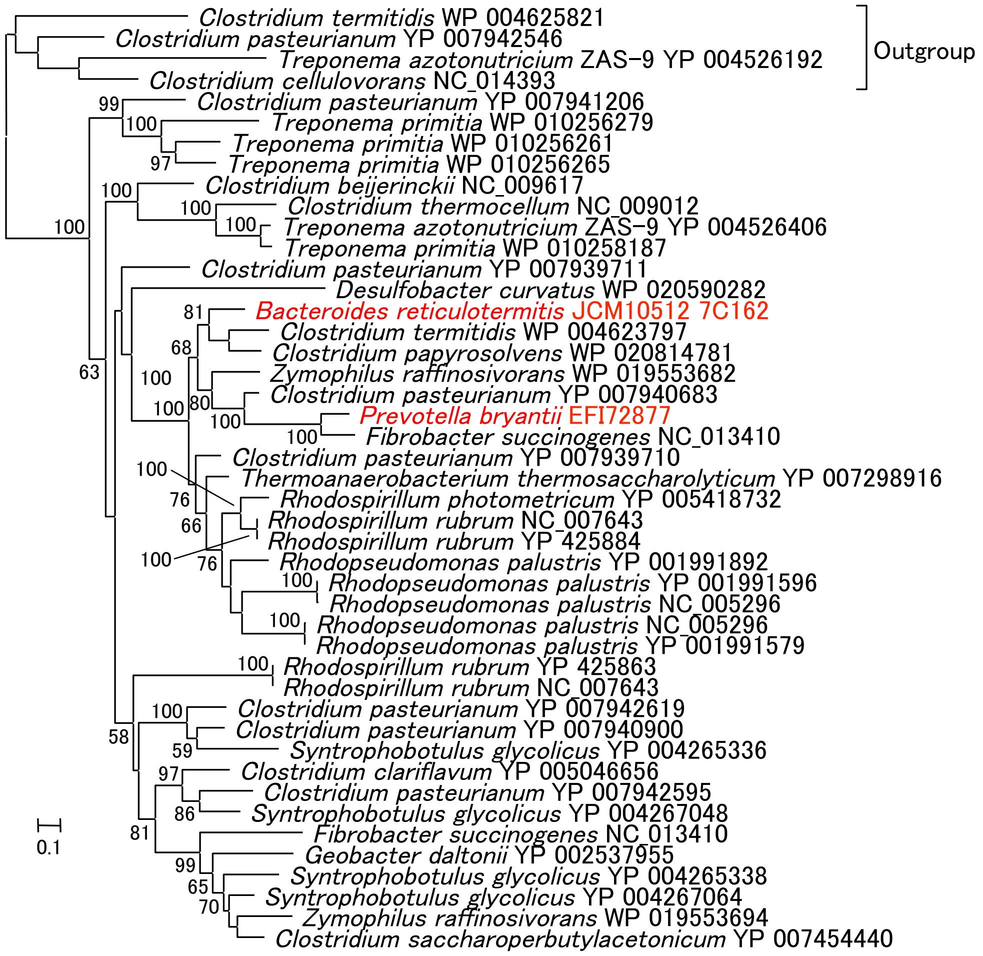

Fig. S8. Maximum likelihood phylogenetic tree of NifN-like sequences. The tree was inferred based on 358 amino acid sites with the selected LG+G+F model. The related sequences in which the iron-molybdenum cofactor ligand Cys 275 (*A. vinelandii* NifD numbering) in the adjacent NifE-like sequence in the genome is absent were used as outgroups. Other details are given in Fig. S7.
